# Higher transactivation activity associated with LTR and Tat elements from HIV-1 BF intersubtype recombinant variants

**DOI:** 10.1186/1742-4690-3-14

**Published:** 2006-02-16

**Authors:** Gabriela Turk, Mauricio Carobene, Ana Monczor, Andrea Elena Rubio, Manuel Gómez-Carrillo, Horacio Salomón

**Affiliations:** 1 National Reference Center for AIDS, Department of Microbiology, School of Medicine, University of Buenos Aires, Buenos Aires, Argentina

## Abstract

**Background:**

HIV-1 is characterized by its rapid genetic evolution and high diversity as a consequence of its error-prone reverse transcriptase and genetic recombination. This latter mechanism is responsible for the creation of circulating recombinant forms (CRFs) found in nature. Previous studies from our lab group have shown that the epidemic in Argentina is characterized by one highly prevalent circulating recombinant form, CRF12_BF, and many related BF recombinant forms. Since transcriptional transactivation of the HIV-1 long terminal repeat (LTR) promoter element requires the essential viral Tat protein, since these genetic structures underwent recombination in variants widely spread in South America, the aim of this work was to study transcriptional activity associated with the recombinant LTR and Tat elements.

**Results:**

Differential transcriptional activity was measured for the BF recombinant LTR/Tat complex that is present in widely spread viral variants was demonstrated. This analysis demonstrated a higher activity for the BF complex when compared to its B subtype counterpart.

**Conclusion:**

This study indicates structural and functional consequences of recombination events within the LTR promoter and Tat transactivator protein of a naturally occurring HIV-1 recombinant form.

## Background

Human immunodeficiency virus type 1 (HIV-1) is a diploid retrovirus whose genome consists of two RNA molecules per virion. This viral RNA serves as the template for proviral DNA synthesis by the virus-encoded reverse transcriptase (RT) enzyme.

HIV-1 is characterized by its rapid genetic evolution and high diversity, as demonstrated by the large number of different HIV-1 strains isolated around the world. This rapid genetic variation provides the virus with maximum adaptation efficiency and presents serious challenges for chemotherapy and vaccine development against HIV-1 infection.

Major mechanisms that contribute to HIV-1 genetic variation include: a high mutation rate as a consequence of its error-prone reverse transcriptase, and recombination [1,2]. Genetic recombination plays an important role in the evolution of HIV-1 [3]. Since two RNA molecules are packaged into each virion, RT can use portions of each RNA as template during reverse transcription to generate a recombinant viral DNA, redistributing the genetic information. Therefore, rapid recombination of the HIV-1 genome creates a vast advantage for viral evolution and an enormous difficulty for the host to control the infection.

Accumulating evidence has confirmed the existence of recombinant HIV-1 in nature [4]. Some of these recombinant viruses have become fixed in the human population and are referred to as circulating recombinant forms (CRFs), and in a few cases CRFs have become the predominant strain in specific geographic areas [5,6].

Previous studies from our lab group have shown that the epidemic in Argentina is characterized by a circulating recombinant form, CRF12_BF, and many related BF recombinant forms [7-9]. The analysis also showed that HIV-1 BF recombinant viruses have diverse mosaic structures that are phylogenetically related in their F and selected B fragments to the F1 subtype and with BF recombinant viruses from Brazil, respectively [9,10].

After HIV-1 gains entry to its target cell, the virus integrates into its host genome. Once integrated, the provirus serves as a template for transcription of viral genes. Regulation of early events in viral transcription is mediated by direct interaction between cellular transcription factors and cis-acting elements located in the HIV promoter or LTR (Long Terminal Repeat) region. At this early phase, the HIV-1 promoter is under control of local chromatin environment, which determines the basal transcriptional activity (reviewed in [11]). As the viral Tat protein accumulates in the nucleus, it dramatically increases transcriptional rate by modifying chromatin conformation at the integration site, adjusting the activity (initiation and elongation) of RNA polymerase II and promoting NF-κB activation [12-15]. Other mechanisms of Tat-mediated activation of viral transcription have been described [14,16-18].

As both viral elements mainly involved in HIV-1 transcriptional transactivation (Tat protein and the LTR promoter) underwent intersubtype genomic recombination in variants widely spread in South America, the following questions can be addressed: What are the implications of intersubtype recombination on both the Tat protein and LTR promoter sequences and their activities? How may these modifications in transcriptional properties influence viral infectivity? The aim of this work was to reveal possible transcriptional variation due to the LTR and Tat recombinant structures. In this study we report not only structural but also functional consequences of recombination events in the LTR promoter and transactivation protein of the BF recombinant form.

(The research performed by Gabriela Turk was in partial fulfilment of her Ph.D. degree, School of Medicine, University of Buenos Aires, Buenos Aires, Argentina).

## Results

### Structural analysis of Tat protein and LTR promoter from BF samples

#### Primary structure of tat variants from HIV-1 BF intersubtype recombinant forms

**Figure 1:**
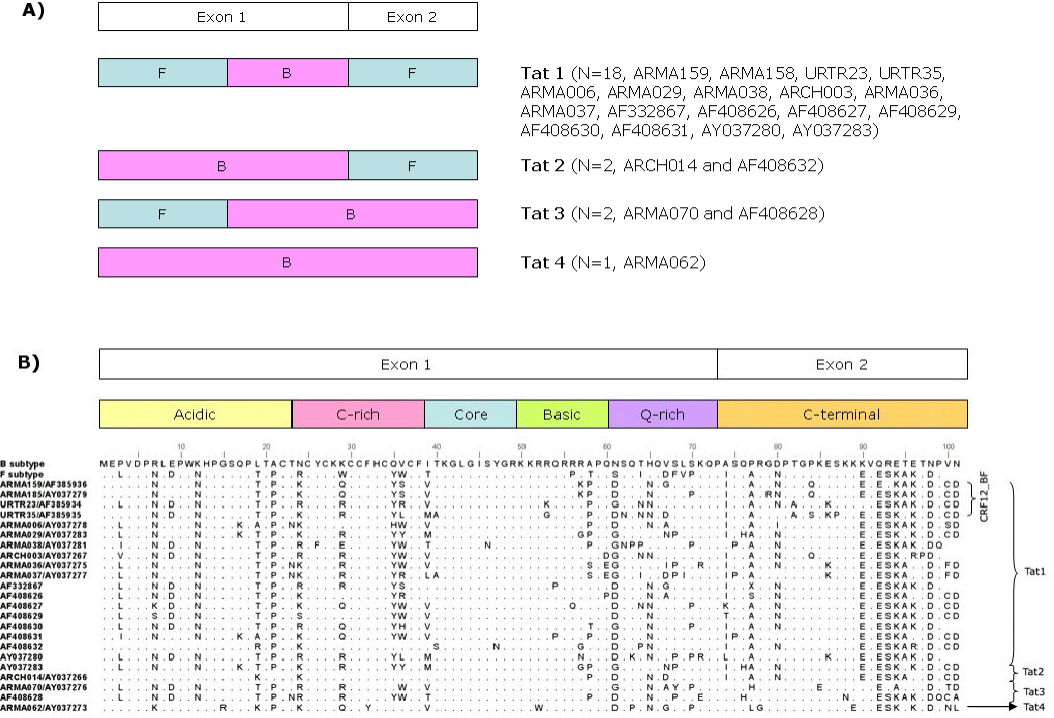
**Tat variants from BF recombinant forms obtained from 23 Argentinean patients**. Samples are named either as ID/accession number (when they were firstly described by our group) or GenBank accession number only. **A. **Four distinguishable genetic patterns (named Tat1, Tat2, Tat3 and Tat4) were recognized by boostcaning analysis. Samples showing each recombination pattern are indicated on the right. **B. **Tat primary structure from Argentinean samples. Several amino acid substitutions are observed when compared to the B subtype prototype sequences. Substitutions are found in every functional domain being the core and basic domains the most conserved.

As it has been reported, BF recombinant forms show different mosaic patterns at the *pol *region [10]. When analyzing the *tat *coding region of previously reported full length sequences corresponding to BF intersubtype recombinant forms, 4 different structures, named Tat1, Tat2, Tat3 and Tat4, were found (Figure 1A). Tat1, the most prevalent variant, was observed in CRF12_BF prototypic strains (ARMA159, ARMA185, URTR23 and URTR35) and in other 14 sequences. In these samples, the *tat *coding region is predominantly F subtype with a B segment in the second half of the first exon. Tat2 and Tat3 are each represented by only 2 sequences (ARCH014, AF408632 and ARMA070, AF408628, respectively). In samples showing the second Tat variant, each exon clustered with a different subtype. Tat3 shares the same BF recombinant exon 1 with Tat1, whereas exon 2 is pure B subtype. Tat4 was only found in ARMA062 and it clustered entirely with the B subtype, showing no recombination breakpoints.

As regards the polymorphisms observed in our samples, we firstly focused on target residues for post-translational modifications (See Figure 1B for primary sequence details). Tat undergoes multiple post-translational modifications, such as acetylation, ubiquitination and methylation, which are responsible for the regulation of its interaction either with RNA and/or cellular factors such as cyclinT1, p300/CBP and PCAF [19]. Our analysis revealed that most of these target sites are conserved among BF viral variants circulating in our country. For instance, K28, K50 and K51 have been widely described as targets for PCAF and p300 acetyltransferases and they are crucial for transcriptional activation. These residues are highly conserved in all the sequences analyzed here, pointing out the importance of these residues for optimal Tat activity. K71, a residue whose ubiquitination results in a non-proteolytic mechanism controlling Tat activity [20], is also highly conserved among all our samples but 3 (ARMA036, AF408628 and AY037280), which showed substitutions to either arginine (R) or glutamic acid (E). Finally, Tat is also substrate for arginine methylation at its basic domain. This domain spans residues 49 to 57, it is composed of 6 Rs and includes K50 and K51. Residues at positions 49 and 55 are conserved in all the analyzed samples meanwhile R52, R53, R56 and R57 are conserved in all but 1, 2, 1 and 5 samples, respectively. It is worth noting that all these substitutions are shown in different samples, i.e. none of the samples have more than one simultaneous R substitution at the R rich motif. Another interesting feature is that the most polymorphic residue at the basic domain is R57 which is the most distant residue from K50. It may be hypothesized that residues closer to K50 are important for Tat biological activity while the others are more dispensable.

Another noteworthy polymorphism was found at K29. Substitutions in this position were present in 19 out of 23 analyzed samples, mostly to R or glutamine (Q). Eleven out of 19 samples showed a simultaneous change at position 24 (N to K) while the remaining 8 showed an N to R change.

As regards Tat domains, the N-terminal or acidic domain is predicted to form an α-helix [21]. In addition to the negatively charged amino acids placed in this domain, B subtype Tat has 2 positively charged residues (R7 and K12) that are likely to stabilize the secondary structure. Twenty out of 23 BF samples showed asparagine (N) substitutions in one (n = 2) or both (n = 18) residues. As N has no charge at physiological pH, these substitutions change the net charge of the whole domain. When analyzing the core and basic domains, no major changes were observed.

The C-terminal domain (aa 73–101), encoded by the second exon of the gene, shows a considerable variability among different viral strains and only a few biological activities have been attributed to it. The first functional motif identified in this domain was the RGD motif which is involved in integrin-mediated cell adhesion [22]. Only 7 of the samples examined here-in preserved this motif intact where most of them showed mutations to RGN. It was also observed that motif ESKKKVESKA is strikingly conserved among Argentinean sequences, although the functional significance of such motif has not been examined in detail yet. This amino acid sequence is present in the F subtype Tat but is not present in the B clade Tat.

#### LTR sequences from BF recombinant variants

**Figure 2:**
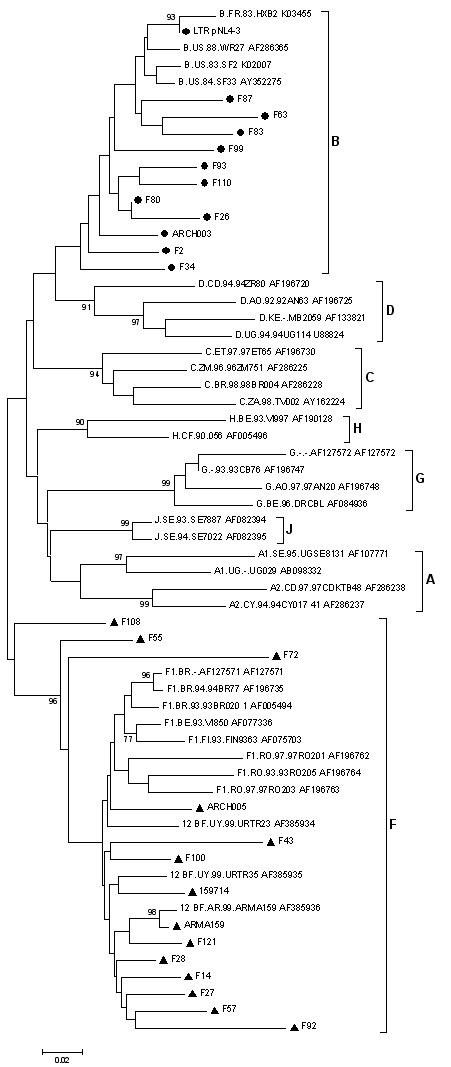
**Phylogenetic analysis of 24 LTR sequences from Argentinean samples**. A neighbour-joining phylogenetic tree was built and maximum parsimony bootstrap values were calculated. The genetic distance corresponding to the length of the branches is shown by the bottom line. Reference samples are those recommended by the Los Alamos Database. ▲ and ●: Unknown samples.

DNA samples previously characterized as BF intersubtype recombinant forms at the *vpu *locus (F02 to F121), obtained from newly infected individuals between 2003 and 2004, were used as template for LTR amplification (HXB2 nt -327 to 179). Direct sequencing was performed on the PCR products, and subtyping was performed by aligning with reference sequences from different subtypes. The population-based phylogenetic analysis showed that 11 out of 24 samples clustered together with B subtype references, and the remaining 13 clustered with F subtype references, confirming its BF recombinant nature (Figure 2). Distance scanning and bootscanning analysis were performed for all samples. This analysis further confirmed the information given by the phylogenetic tree, even for samples ARCH003, F2, F34, F55, F72 and F108 that seemed to be less phylogenetically related to their corresponding clusters (data not shown).

**Figure 3:**
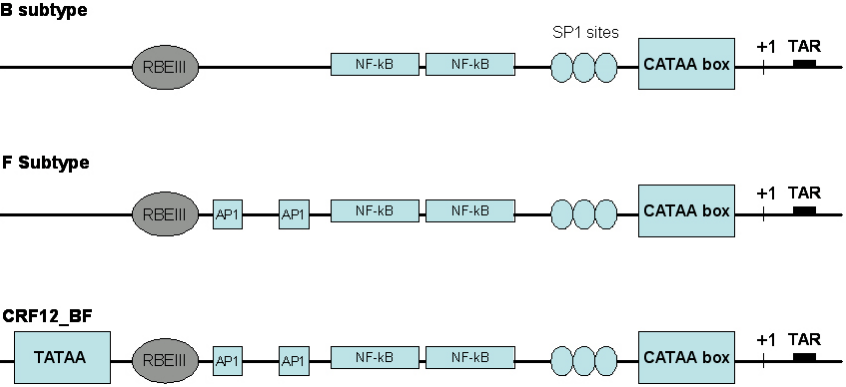
**LTR maps for transcription binding sites**. Organizations of HIV-1 subtype B, F and CRF12_BF recombinant LTRs are shown. Subtype B has been used as a reference for protein binding sites.

To investigate the presence of transcription factor bindings sites (TFBS) in these recombinant LTRs, sequences were analyzed using the TFSEARCH program (available at ). Major TFBS were included in this analysis, i.e. USF, TATA box, RBEIII, SP1, AP1 and NFκB (Figure 3). Sample ARMA 159, prototype of the CRF12_BF, showed an additional TATAA box located at position -136. Also sample F121 turned out to have the additional TATAA sequence. It is remarkable that both sequences, F121 and ARMA 159, clustered closely in the phylogenetic tree (see figure 2). This additional TATAA sequence has also been reported for subtype E LTR and it has been shown as functionally inactive in this subtype [23]. van Opijnen *et al *have also shown that a CATAA box is the actual regulatory sequence at the LTR promoter, and it was present in all the samples analyzed here [23].

RBEIII is a cis-acting element known to be binding site for RBF-2, the Ras response element binding factor 2, recently described as the USF1/USF2 heterodimer [24]. This site was found in duplicate in only 1 of the 24 samples studied (F14). Even though this duplication has been described previously [25,26] and it is one of the most frequently occurring polymorphism of the HIV-1 LTR, its clinical implications are still not clear. Estable *et al *suggested that this polymorphism would be selected *in vivo *because it could down-regulate the HIV-1 transcription during monocyte to macrophage differentiation or T-cell activation [25]. On the other hand, no significant differences were found in number and structure of USF, SP1, AP1, and NFκB sequences.

**Figure 4:**
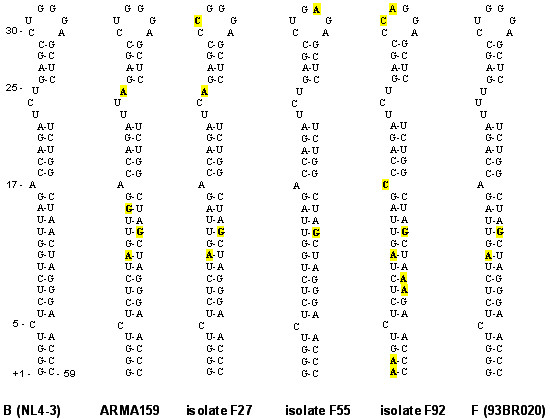
**TAR structure of BF recombinant variants**. TAR secondary structure of CRF12_BF (ARMA159), and BF recombinant isolates F27, F55 and F92 are shown. B (NL4-3) and F (isolate 93BR020) subtype TARs were used as reference. Nucleotide changes are highlighted. Changes were found at positions 31 (U31), 32 (G32) and 33 (G33) in these isolates. Nucleotide position 32 is one of the essentials for CycT1-Tat interactions with TAR RNA. Nucleotides U31 and G33 are not critical, but they contribute to the stabilization of the RNA-protein complex.

The TAR RNA element is positioned immediately after the transcription start site (nt +1 to +59) while forming a stable hairpin structure, and having a wide natural variation that has been described previously [27]. This element was analyzed and changes in relevant nucleotide positions in the loop region were found (Figure 4). Nucleotides in this loop have been shown to be essential in the Tat-CyclinT1/CDK9 interaction for activating transcription elongation from HIV-1 LTR. Changes in this region (nucleotides 30–35) have been related to variations in the interaction with CyclinT1 while changes at positions 23–24 (bulge region) have been shown to affect the interaction with Tat [28]. Four of the analyzed samples (F27, F55, F67 and F92) presented changes in the highly conserved 6-nucleotides sequence (CUGGGA) and changes located at positions 23–24 that form another loop in the TAR stem. Changes were found at positions 31 (U31), 32 (G32) and 33 (G33). Nucleotide positions 32 and 34 in the loop region are essential for CycT1-Tat interactions with TAR RNA. The identity of nucleotides U31 and G33 is not critical, but they contribute to the stabilization of the RNA-protein complex.

### Functional analysis of of Tat protein and LTR promoter from BF samples

#### GFP transactivation in GHOST cells

**Figure 5:**
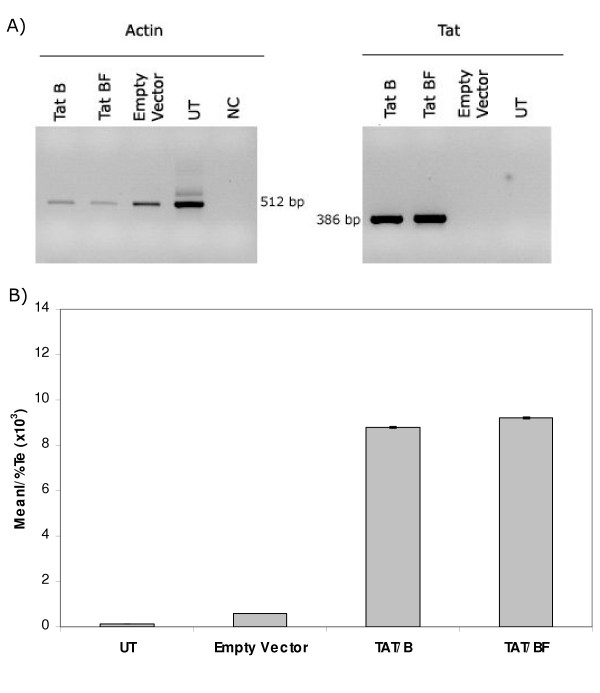
**Transactivation of GFP expression in GHOST cells. **A) *tat *mRNA was detected in pTat transfected cells, but not in untransfected cells or cells transfected with empty vector, by RT-PCR (right panel). Amplification of actin mRNA was also evaluated as an internal control (left panel). B) Tat expressing vectors were capable to transactivate the expression of the *gfp *gene placed downstream the LTR from HIV-2. No statistical difference was found between Tat^B(NL4-3) ^and TAT^BF(ARMA159)^. UT: Untransfected cells, NC: Negative control, MeanI: Mean Fluorescence Intensity, Te: Transfection efficiency. Data presented here is representative of 4 independent assays.

With the purpose of evaluating both protein expression and activity of our full length *tat*-coding constructs, GHOST cells were transiently transfected with pTAT^BF(ARMA159) ^and pTAT^B(NL4-3)^. Forty-eight hours post-transfection *tat *mRNA was detected by using a qualitative RT-PCR (Figure 5A). As regards Tat biological activity, it was invariably found (after four independent assays, each one in triplicate) that there is no significant difference between Tat^B(NL4-3) ^and Tat^BF(ARMA159) ^to transactivate the expression of an HIV-2 LTR-driven reporter gene (Figure 5B).

#### Tat capability to restore viral transactivation in HLM1 cells

**Figure 6:**
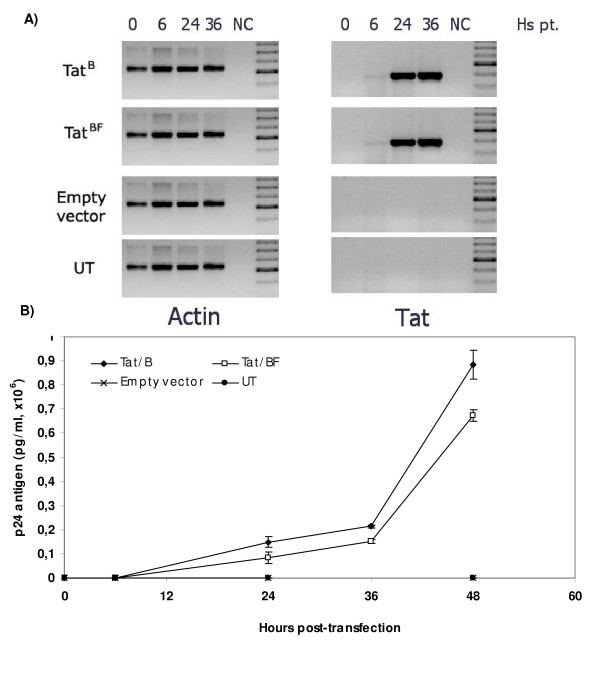
**Restoration of viral production in HLM1 cells**. A) *tat *mRNA was detected in pTAT transfected cells as early as 6 hours post-transfection (right panels). Amplification of actin mRNA was used as an internal control (left panels). B) Kinetic of p24 production after cell transfection with Tat encoding vectors. UT: Untransfected cells, NC: Negative control, Hs pt: Hours post-transfection. Data presented here is representative of 3 independent assays.

In order to evaluate if Tat derived from a pure HIV-1 subtype or a prototypic recombinant form share the same ability to transactivate viral production, HLM1 cells were either pTAT^BF(ARMA159) ^or pTAT^B(NL4-3) ^transfected. Viral production was evaluated at several time points after transfection in cell culture supernatants. Qualitative RT-PCR showed that *tat *mRNA was readily detected as early as 6 hs post transfection (Figure 6A). However, viral production was not evident until 12 hs post-transfection (not shown). Up to 36 hs post-transfection, Tat^B(NL4-3) ^– and Tat^BF(ARMA159) ^– driven viral transactivation reached similar levels (Figure 6B). However, at this time point, it was consistently seen that Tat^B(NL4-3) ^tended to show higher transactivation levels (although non-significant). In agreement with this observation, 48 hs post-transfection it was observed that Tat^B(NL4-3) ^showed a significantly improved capacity to drive viral production (*p *= 0.004).

#### Transcriptional levels of BF recombinant LTRs

**Figure 7:**
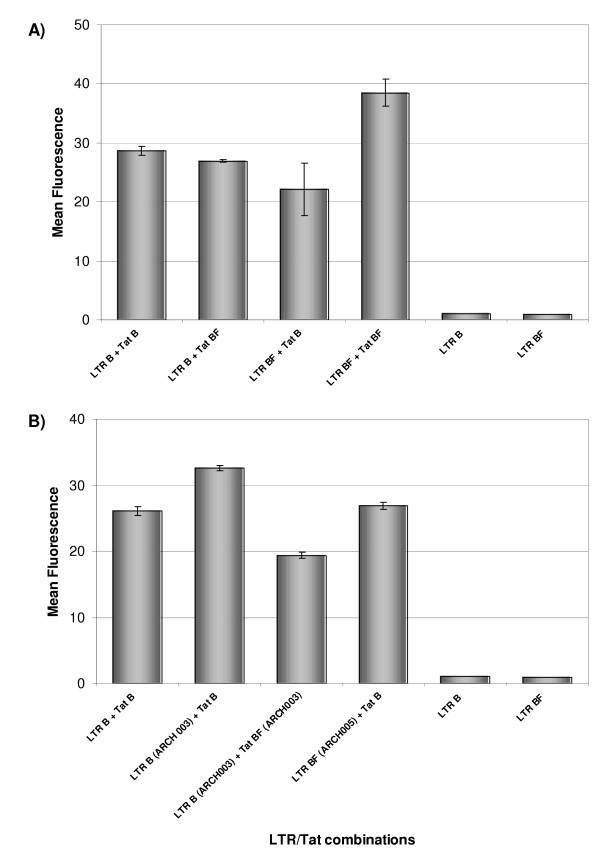
**Transactivation of B and BF LTRs by clade B and BF specific Tat proteins (A: CRF12_BF; B: ARCH003 and ARCH005)**. 293T cells were transfected with GFP reporter constructs containing HIV-1 clade-specific LTRs in absence or presence of expression plasmids encoding Tat ^B(NL4-3) ^or Tat^BF(ARMA159)^. Relative transactivation is expressed as mean fluorescence obtained by FACS analysis. Cells transfected only with LTRs constructs were used as negative controls. Error bars indicate standard errors.

Previous study has shown that LTRs from different subtypes have slightly different transcriptional capacities [29,30]. By co-transfecting 293T cells with plasmids containing either the LTR^B(NL4-3) ^or LTR^BF(ARMA159)^, and plasmids pTat^B(NL4-3) ^or pTat^BF(ARMA159) ^their transcriptional activities were tested and compared. FACS analysis, performed 48 hs after transfections, showed an increased transcriptional level associated to the BF recombinant LTR when compared to the subtype B LTR (*p *= 0.02). This difference was evident when pLTR^BF(ARMA159) ^plasmid was co-transfected with pTat^BF(ARMA159) ^(LTR^BF(ARMA159) ^/Tat^BF(ARMA159) ^vs. LTR^B(NL4-3)^/Tat^B(NL4-3) ^and LTR^BF(ARMA159) ^/Tat^B(NL4-3) ^vs. LTR^BF(ARMA159) ^/Tat^BF(ARMA159)^). Slightly but not significant differences in transcriptional levels were observed when LTRs were co-transfected with a Tat of different subtype (LTR^BF(ARMA159) ^/Tat^B(NL4-3) ^vs. LTR^B(NL4-3)^/Tat^BF(ARMA159)^) (Figure 7).

ARCH003 and ARCH005 are samples obtained from vertically infected children whose genetic structure suggested the circulation of the BF recombinant forms since the mid-1980's [31]. ARCH003 has a B subtype LTR and a BF recombinant structure in the *tat *coding region while ARCH005 has a BF-like LTR and Tat structures. When LTR transcriptional activity from ARCH003 sample was analyzed it turned out to be higher when it was transactivated by the B subtype Tat protein than when its own BF Tat protein (pTat^ARCH003^) was used (*p *= 0.001). ARCH005 LTR was only evaluated using the B subtype Tat and it did not show significant differences when compared to the LTR^B(NL4-3)^/Tat^B(NL4-3) ^complex. Unfortunately, the *tat *region from this sample could not be amplified.

## Discussion

This study characterizes the genetic structure of LTRs sequences and Tat proteins from BF recombinant variants and, also, analyzes the transcriptional activity associated with these recombinant variants.

LTR diversity among different HIV-1 subtypes may affect the binding of both cellular and viral transcription factors, thus influencing the transcription level. This effect, in turn, may have biological consequence for the different HIV-1 clades. Although studies on LTR transcriptional activity of different HIV-1 subtypes have been previously performed [23,29,30], the consequences of intersubtype recombination on HIV-1 transcriptional activity has not been analyzed in much detail. Moreover, most studies that involved the analysis of LTRs from different subtypes always used the B subtype Tat protein as transactivator, which hampers the proper evaluation of the results in terms of transcriptional advantages, due to sequence changes in Tat protein activity due to recombination events. In this *in vitro *study, a differential transcriptional activity associated to the BF recombinant LTR/Tat complex found in widely spread viral variants in Argentina was shown. This analysis demonstrated a higher activity for the LTR^BF(ARMA159) ^/Tat^BF(ARMA159) ^complex when compared to its B subtype counterpart. It also showed that LTR and Tat proteins from different subtypes (B vs. BF) rendered a lower activity when Tat and LTR did not match in their subtype, i.e. LTR^B(NL4-3)^/Tat^BF(ARMA159) ^and LTR^BF(ARMA159) ^/Tat^B(NL4-3)^. Phylogenetic analysis of LTR sequences from the prototypic CRF12_BF sample (ARMA159) demonstrated that it clustered together with F reference sequences. The improved transcriptional levels measured for BF recombinant LTRs are consistent with previously reported high activity of the F subtype LTR [29].

Several substitutions were found in the *tat *gene and in the LTR sequence from BF samples when compared to their B subtype counterparts, which may account for the improved transcriptional potential observed here. When the Tat sequence was analyzed in detail, it was found that previously reported elements considered to be crucial for Tat activity were conserved among BF viral variants thus stressing the key role of K28, K50, K51 and the basic and core domains in transactivation. These 2 regions are particularly important for Tat function as they are involved in cyclin T1 binding, nuclear localization, Tat-mediated pro-apoptotic-effects and interaction with a wide subset of transcriptional coactivators and modifying enzymes [16-18]. More recently, it was found that Tat is also able to abrogate RNA silencing by inhibiting the ability of DICER to process precursor double-stranded RNAs into siRNAs. Authors demonstrate that K51 is a key residue for Tat suppressor of RNA silencing activity [32].

Previous publications describing HIV-1 gene expression driven by clade-specific Tat proteins found that Tat proteins from E and C subtypes are the most active in transactivation [33,34]. Higher transactivation potential of subtype E Tat was attributed to its longer half-life, which in turn, was postulated to be dependent on its ubiquitination pattern [33]. Since the E subtype Tat protein contains more lysine residues acting as ubiquitin acceptor, ubiquitination could occur more readily and contribute to its extended half-life. Most BF Tat variants (14 out of 23), including CRF12_BF prototypic strains have a lysine at position 24 instead of the asparagine residues that characterize B subtype isolates. K24 is a shared feature with the E subtype Tat and, although speculative, this may partially account for the higher activity observed for Tat BF.

In order to assess the transactivating capacity of Tat^B(NL4-3) ^vs. Tat^BF(ARMA159) ^proteins we used GHOST cells as a reporter system and also HLM1 cells to observe restoration of viral production. In GHOST cells, we were not able to observe differences between Tat^B(NL4-3) ^and Tat^BF(ARMA159)^. As reporter gene expression in these cells is driven by the LTR promoter from HIV-2, it is possible that both proteins, although structurally distinct, share the same mechanism to modulate transcription in this system. In HLM1 cells, Tat^B(NL4-3) ^showed an improved ability to rescue viral production from the Tat-defective provirus (which harbours a B-matched LTR). This is consistent with the results obtained from the co-transfection assays where the LTR^B(NL4-3)^/Tat^BF(ARMA159) ^combination had the lowest transcriptional potential. A naturally occurring LTR^B(NL4-3)^/Tat^BF(ARMA159) ^combination was observed in sample named ARCH003. Transmission of this viral variant can be tracked back to the year 1986. It was isolated from a vertically infected child with no evidence of reinfection. *In vitro *co-transfection assays showed that the LTR^ARCH003^/Tat^ARCH003 ^complex had significantly lower transactivation potential when compared to other LTR/Tat combinations but when LTR^ARCH003 ^was co-transfected with Tat B, higher expression of the reporter gene was obtained. According to the extensive molecular epidemiology data in our region, we suggest that early BF recombinant forms had a recombination breakpoint at the Vpu/Tat region but conserved the LTR of B subtype. This would render the emerging variants less competent for transcriptional activation, and natural selection would have forced the gaining of an F-like LTR. In other words, it is possible to speculate that F subtype sequences present in the BF recombinant would be advantageous for the virus since these changes would increase its fitness, in terms of transcription. Nevertheless it is not clear yet if this advantage correlates with what is observed in the field of the epidemiology.

The LTR analysis showed moderate diversity between the sequences analyzed. An extra TATA box was found in CRF12_BF sample and in other patient isolate (F121). Although this extra sequence is postulated to be inactive in other HIV subtype, i.e. E subtype, *in vitro *studies on this issue are currently in progress. Nucleotide changes were also found in the TAR region. This region is important for the Tat/TAR/cellular factor interactions implicated in the activation of transcription. Base changes at positions 31, 32 and 33 of the loop may affect the interaction with proteins Cyclin T1 and CDK9. Major TFBS appeared to be conserved between samples with the exception of RBEIII, which was found in duplicate in one sample. This factor is important for transcriptional silencing in resting infected cells and this is the most common polymorphism in clinical isolates. These changes could be, at least in part, related to the differential transcriptional activities observed in the BF recombinant variants.

## Conclusion

In summary, data presented here shows transcriptional differences linked to the LTR and *tat *coding region of BF recombinants circulating in Argentina. Transcriptional improvement may be a consequence of the recombination process between 2 different HIV-1 subtypes and selection forces favouring the spreading of these recombinant forms. The fitness of BF primary isolates is currently being evaluated in our laboratory. Preliminary results indicate a certain replicative advantage of BF over B isolates, in dual infection-competition assays (Rubio AE *et al*, manuscript in preparation). The correlation between these *in vitro *data and the patients' clinical outcome is still not clear due to the lack of follow-up studies in BF infected patients. Studies on viral load set-point, disease progression as well as transmissibility of BF variants will be helpful to estimate the impact of emerging recombinant HIV-1 genotypes on the spreading epidemic.

## Methods

### DNA samples and amplification

Extensive HIV-1 genotyping studies have been conducted by our group. Genomic DNA from HIV-1 infected patients involved in these studies was used to amplify the LTR region and *tat *full coding sequence. Samples previously characterized as BF intersubtype recombinant forms at the *vpu *locus (F02 to F121) were obtained from newly infected individuals between 2003 and 2004. ARMA159 is the prototypic strain for CRF12_BF (GenBank accession N° AF385936; [9]), ARCH003 and ARCH005 (GenBank accession N° AY037267 and N° AF454487, respectively) were obtained from children born to HIV-1 infected mothers. Full length and partial sequences of ARCH003 and ARCH005 samples are available, respectively. Both samples show recombination between B and F subtypes although ARCH005 seems to be closely related to CRF12_BF [31]. HIV-1 molecular clone pNL4-3 was used as B subtype template. LTR region (HXB2 nt -327 to 179) and first and second Tat exons were amplified by nested PCR. All reactions were conducted using the proof-reading Vent DNA polymerase (New England Biolabs, USA). Primers are listed in Table 1. Pasting of *tat *exons was performed in one further PCR round using primers TECORI and TXHOI. This allowed us to place both exons together in frame in one final amplicon.

**Table 1:** List of primers used for PCR amplification

**Target region**		**Primer name (sequence 5'-3')**
LTR	1R	F: LTR1 (cacacaaggctayttcctga) R: JL17 (cattctgcagcttcctcattgat)
	2R	F: LTR3 (tggatggtgctwcaagytagt) R: LTRrev (tgctagagattttccacactgac)
*tat *1st exon	1R	F: polseq2 (cgggtttattacagggacagc) R: ES33 (cattgccactgtcttctgctc)
	2R	F: TECORI (cagaataggaattctgcgacagagaag) ^*1^ R: TE1 (gggatatgggttgctttgatagagaagc)
*tat *2nd exon	1R	F: KS2mod (ttctatagtggatagagttaggaaggg) R: OFM19mod (gcactcaaggcaagctttattgaggc)
	2R	F: TE2 (gcttctctatcaaagcaacccatatccc) R: TXHOI (acaggctcctcgaggtcgtccc) ^*2^
β-actin		F: β1 (ggacctgactgactacctcatgaa) R: β2 (gatccacatctgctggaaggtgg)

1R = 1st round, 2R = 2nd round. Introduced EcoRI (^*1^) and XhoI (^*2^) restriction sites are underlined. F: Forward. R: Reverse.

### Cloning and sequencing

Constructs named pLTR^B(NL4-3)^, pLTR^BF(ARMA159)^, pLTR^ARCH003 ^and pLTR^ARCH005 ^were generated by cloning the LTR amplicons into the pGLOW reporter vector (Invitrogen, USA), upstream of the GFP reporter gene. Full length *tat *amplicons were cloned into the commercial expression vector pTARGET (Promega, USA) generating pTAT^B(NL4-3)^, pTAT^BF(ARMA159) ^and pTAT^ARCH003^. Colony screening was performed by colony-PCR and restriction analysis. Minipreps of positive clones were obtained (QIAprep Miniprep kit, QIAgen INC, USA) and sequenced using the Big Dye Terminator sequencing kit (Amersham, Sweden) on an automatic sequencer (Applied Biosystems DNA sequencer 3100). Nucleotide sequences were analyzed and manually adjusted using Sequencher 4.0.5 software (Gene Codes Co, USA). Selected constructs were large-scale prepared using the HighSpeed Midiprep kit (QIAgen INC) and quantified in agarose gels.

### Cells and DNA transfection assays

HLM1, GHOST and 293T cell lines were used in this study. HML1 cells are HeLaT4+ cells stably transduced with a *tat*-defective HIV-1 molecular clone and were obtained through the AIDS Research and Reference Reagent Program, Division of AIDS, NIAID, NIH: HLM30 cells from Dr Reza Sadaie [35]. HLM1 cells are negative for virus particle production but they can be induced to express high levels of non-infectious HIV-1 and syncytial cells after transfection or coculture with *tat*-expressing clones. GHOST is a human osteosarcoma (HOS) derived cell line stably transduced with MV7neo-T4 retroviral vector and stably co-transfected with the HIV-2 LTR driving GFP construct (Kewalramani VN, unpublished data). 293T is a highly transfectable kidney-derived epithelial cell line.

Cells were grown at 37°C and 5% CO_2 _in a humidified incubator. 293T cells were cultured in Dulbecco's modified Eagle's medium (DMEM, Gibco BRL, USA) supplemented with 10% fetal bovine serum (FBS, Gibco BRL), 2 mM L-glutamine (Gibco BRL), 100 U/ml penicillin (Gibco BRL) and 100 mg/ml streptomycin (Gibco BRL). GHOST cells were grown in the same medium plus 400 μg/ml geneticin (Gibco BRL). HML1 cells were cultured in DMEM supplemented with 10 μg/ml gentamicin (Gibco, BRL) and 10% FBS.

HLM1, GHOST and 293T cells transient transfections were carried out using Lipofectamine2000 (Invitrogen), following manufacturer instructions. For single transfections experiments confluent cells grown in 60 mm Petri dishes were transfected with 8 μg of the corresponding pTAT construct. For 293T co-transfections, 5 μg of pLTR and 0.5 μg of pTAT were used. Untransfected cells as well as cells transfected with empty vectors were always used as negative controls. Also, empty vectors were used to keep DNA amount constant in dual transfection experiments.

### RT-PCR

Total cellular RNA was extracted from 10^7 ^cells using Trizol reagent (Gibco BRL). Three μg of RNA were reverse transcribed using MMLV reverse transcriptase (Invitrogen) and an oligo-dT primer. The mix was supplemented with RNAse inhibitors (Invitrogen) in a final volume of 20 μl. Two μl of cDNA were used for *tat *PCR amplification using TECOI and TXHOI primers. Amplification of β-actin was used as RNA quality control. Actin PCR was also performed on 2 μl of the RT reaction using primers β1 and β2-actin (Table 1).

### GFP transactivation in GHOST cells

The use of the green fluorescent protein (GFP) gene as a reporter molecule has been widely used to study the transcriptional activity of the HIV LTRs from different isolates. The fluorescence intensity of GFP is a direct measurement of the transcriptional activity of the promoter that directs the expression of the protein. Upon infection or transfection of cultured cells with a Tat coding plasmid, GFP expression can be measured using a fluorescence activated cell sorter. One of the main advantages in using this system is that it is possible to simultaneously measure transfection efficiency and fluorescence intensity of the transfected live cells without the need of co-transfection of a reference plasmid [36,37].

Tat constructs were used for single transfections of GHOST cells. Expression of the reporter gene was analyzed by flow cytometry (FACS Coulter Epics XL, Becton Dickinson, USA) in cells harvested 48 hs post-transfection. Living cells were gated by forward angle and side-scatter light. A minimum of 10,000 events were collected for each histogram. Analytical gates were set such that 1% or fewer of negative control cells fell within the positive regions. Data analysis was carried out using System II software (Beckman Coulter INC, USA). Four independent assays were conducted, each in triplicate. Data was expressed as the Mean Fluorescence Intensity (MeanI)/% of GFP positive cells ratio.

### Restoration of transactivation in HLM1 cells

Tat constructs were used for single transfections of HLM1 cells. Cell culture supernatants were collected 6, 12, 24, 36 and 48 hs post-transfection in order to evaluate viral production. p24 titers were subsequently determined through a commercial ELISA assay (Murex, Abbott, USA) including a calibration curve. Three independent assays were conducted, each in triplicate.

### Subtype-specific LTR transactivation assays

293T were double transfected with different pLTR and pTat constructs. Expression of the reporter gene driven by the interaction between Tat and the corresponding LTR sequence was analyzed by flow cytometry as described above.

### Statistical analysis

All data was expressed as mean ± SD unless, otherwise stated. Significance (*p *< 0.05) between means of two experimental groups was evaluated using the Student's *t *test for independent samples (through the Primer of Biostatistics version 4.02 software).

### Phylogenetic and TFBS analysis

A multiple alignment of the newly generated LTR sequences with selected reference sequences was performed using ClustalX, and visually corrected with the BioEdit version 5.0.9 . Subtype ascribing of LTR sequences was performed by constructing phylogenetic trees by Neighbor-joining using the Kimura 2-parameter model with the MEGA v.3.0 program . Bootstrap analysis was done to assess the stability of the nodes. Recombinant analysis was performed by bootscanning by using the SimPlot v.2.5 program  and visual inspection of alignments were used in order to identify breakpoints in recombinant sequences. After identification of the breakpoints, sub-regions of the alignment were re-analyzed by neighbor-joining with bootstrapping to confirm the subtype assignment. Reference sequences included in the analysis were those recommended by the Los Alamos HIV sequence database .

LTR sequences were also analyzed in search of transcription factors binding sites (TFBS) using a web-based method (TFSEARCH Data Base, ).

## Competing interests

The authors declare that they have no competing interests.

## Authors' contributions

GT and MC contributed equally to this work designing and performing the experiments and during the manuscript preparation. AM provided help with LTR amplification and cloning. AER participated during the experimental design and provided technical help. MGC helped with phylogenetic analysis and data interpretation. HS supervised experimental design and writing of the manuscript. All authors read and approved the final manuscript.
